# Balloon-expandable Myval valve-in-valve transcatheter aortic valve implantation with bailout left main coronary artery chimney stenting: a case report

**DOI:** 10.1186/s13256-025-05471-0

**Published:** 2025-08-05

**Authors:** Dibya Ranjan Behera, Kiran Kumar Shetty

**Affiliations:** 1 Manipal Hospital, Plot No.1, Besides Satyasai Enclave Khandagiri, Bhubaneswar, India; 2 Department of Product Performance Engineering & Post Market Clinical Follow Up, Meril Life Sciences, Vapi, Gujarat India

**Keywords:** Transcatheter aortic valve implantation, TAVI-in-TAVI, Aortic stenosis, Degenerated heart valves, Balloon-expandable valve, Myval THV

## Abstract

**Background:**

Transcatheter aortic valve implantation-in-transcatheter aortic valve implantation represents a progressive solution for patients with degenerated transcatheter heart valves, especially those at high surgical risk. With the increasing use of transcatheter aortic valve implantation worldwide, the need for redo procedures is also rising. Balloon-expandable valves such as the Myval transcatheter heart valve (Meril Life Sciences Pvt. Ltd.) offer design advantages of enhanced radial strength, low-profile frames, and effective sealing, making them suitable for complex valve-in-valve scenarios. An essential procedural concern in redo transcatheter aortic valve implantation is the risk of coronary obstruction, particularly involving the left main coronary artery, requiring pre-emptive planning strategies such as the chimney technique.

**Case presentation:**

We report the case of a 68-year-old female patient of Indian ethnicity with prior transcatheter aortic valve implantation using a 26 mm CoreValve (Medtronic), presenting with symptomatic valve degeneration. Her case having been deemed high-risk for open surgical intervention, she was selected for a transcatheter aortic valve implantation-in-transcatheter aortic valve implantation procedure. Preprocedural computed tomography imaging showed a critical risk plane for the left main coronary artery, necessitating coronary protection. A 23 mm balloon-expandable Myval transcatheter heart valve was implanted using transfemoral access. Coronary protection was initiated with prepositioning of a coronary guidewire in the left coronary artery. Following valve deployment, the patient developed hypotension with left main coronary artery flow compromise, requiring bailout left main coronary artery stenting, resulting in a chimney configuration. Postdeployment angiography confirmed optimal valve positioning with preserved coronary flow. The patient initially developed hypotension and severe hypokinesia, which was managed successfully with emergency left main coronary artery stenting and supportive care. She exhibited immediate hemodynamic recovery and was discharged in a stable condition. This is the first documented case of a Myval-based transcatheter aortic valve implantation-in-transcatheter aortic valve implantation with left main coronary artery chimney stenting from East India.

**Conclusion:**

This case highlights the feasibility, safety, and procedural effectiveness of the Myval balloon-expandable transcatheter heart valve in a redo transcatheter aortic valve implantation setting. It also underscores the importance of anatomical evaluation, risk stratification, and pre-emptive coronary protection in complex structural heart interventions. The successful use of the chimney technique reinforces its role in mitigating life-threatening coronary occlusion during valve-in-valve procedures. This report contributes to the growing evidence supporting the use of next-generation transcatheter heart valves for complex redo transcatheter aortic valve implantation scenarios and expands the clinical applicability of Myval in high-risk cases.

## Introduction

Transcatheter aortic valve implantation (TAVI), or transcatheter aortic valve replacement (TAVR), is an effective and cost-efficient therapeutic option for patients with severe aortic valve disease at intermediate to high surgical risk. It offers a less invasive surgical aortic valve replacement (SAVR) alternative. Despite being a relatively recent advancement, TAVI has shown significant progress, with notable reductions in morbidity and mortality. While most procedures are successful and free of complications, suboptimal outcomes can occur in some cases, particularly in the presence of significant aortic regurgitation following transcatheter heart valve (THV) deployment [1].

It is estimated that approximately 1.4–2.8% of patients undergoing transcatheter heart valve (THV) implantation may require a second THV due to significant prosthetic degeneration. Notably, over 70% of these TAVI-in-TAVI procedures are expected to be successful. Performing a subsequent TAVI offers a validated and promising therapeutic option for patients with degenerated index THVs following an initial TAVI. Moreover, the TAVI-in-TAVI approach contributes to refining procedural techniques optimizing preprocedural assessment and postoperative management. It provides critical insights into areas requiring future advancements in TAVI technology [2].

## Case presentation

A 68-year-old female patient of Indian ethnicity presented with a 6-month history of dyspnea, which had worsened over the past month. She also reported severe generalized weakness for 15 days and bilateral lower limb swelling for 3 days. Her medical history was significant for severe aortic stenosis, type 2 diabetes mellitus, and hypertension. In 2017, she had undergone transcatheter aortic valve implantation (TAVI) with a 26 mm self-expanding CoreValve (Medtronic).

### Examination

On clinical examination, the patient was conscious, oriented, and afebrile. Vital signs were stable, with a pulse rate of 69 beats per minute, blood pressure of 110/90 mmHg, and a respiratory rate of 18 breaths per minute. Oxygen saturation was within normal limits on room air. Transthoracic echocardiography revealed a thickened aortic valve with associated left ventricular hypertrophy and a preserved left ventricular ejection fraction (LVEF) of 55%. Mitral annular calcification was also noted. Doppler evaluation demonstrated a peak/mean transvalvular gradient (PG/MG) across the aortic valve of 88/56 mmHg, suggesting significant prosthetic valve dysfunction.

### Preprocedural planning based on computed tomography images

The left coronary artery (LCA) risk plane from the CoreValve inflow was measured at 19.6 mm, while the right coronary artery (RCA) risk plane was 23.2 mm. A 23 mm balloon-expandable Myval transcatheter heart valve (THV) was selected on the basis of anatomical and hemodynamic considerations. Bench testing data for the Myval 23 mm valve indicate a crimped-to-expanded diameter profile of 23–17.85 mm, respectively, with a closed cell height of 8.39 mm in the expanded state.

Bench data informed optimal alignment of the Myval within the existing CoreValve to minimize the risk of LCA obstruction, guiding the decision to position the outflow at node 4. Given the potential discrepancy between bench testing and *in vivo* heavily calcified leaflets, prophylactic LCA protection with a coronary guidewire was employed to ensure safe coronary perfusion during the TAVI-in-TAVI procedure.

### Procedure

The procedure commenced with ultrasound-guided femoral arterial access to ensure precise puncture. A guidewire was advanced into the left anterior descending artery (LAD) to secure coronary access in anticipation of potential interventions. Simultaneously, a pigtail catheter was positioned in the left ventricle (LV) to serve as a radiographic landmark.

**Fig. 1:**
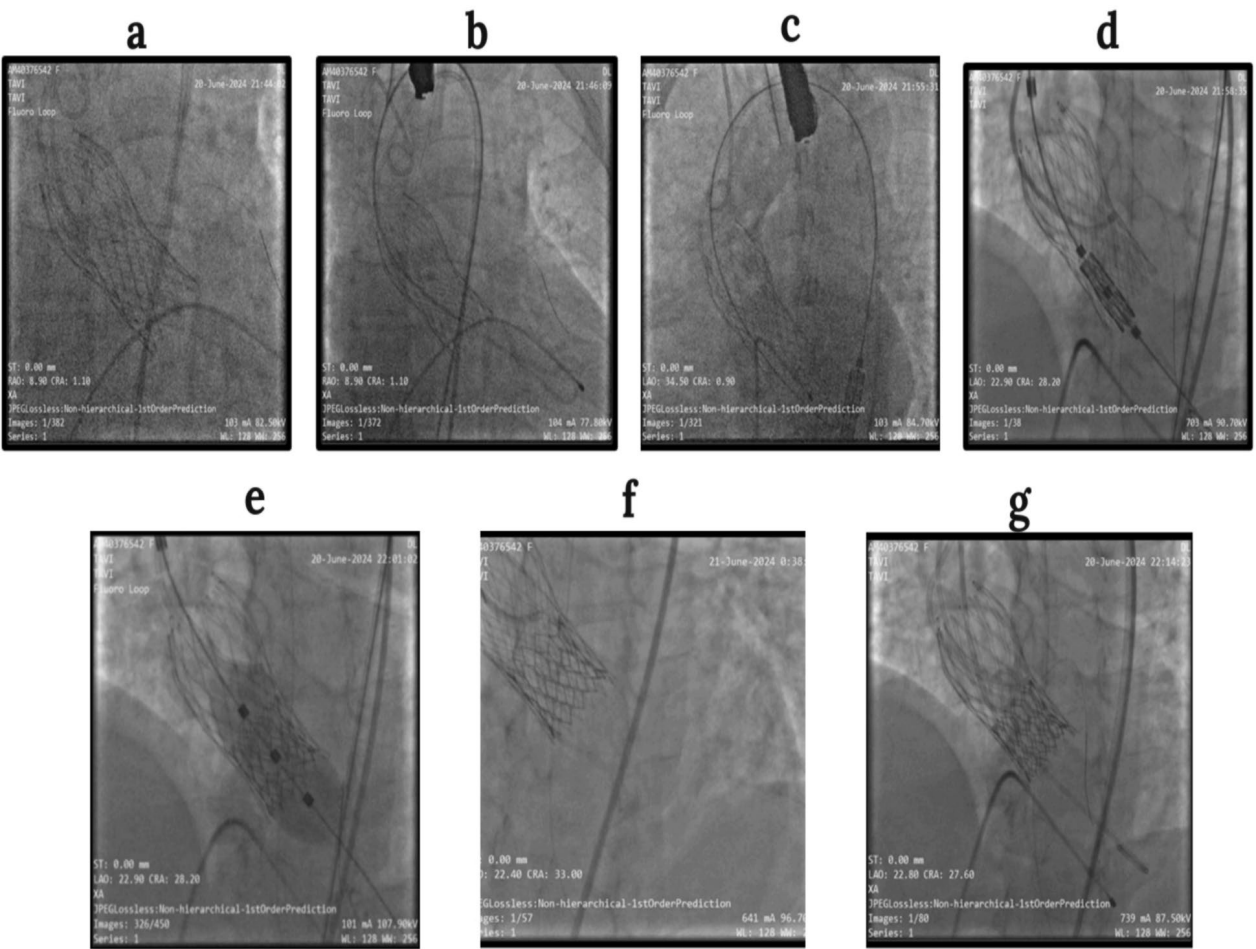
Transcatheter aortic valve implantation-in-transcatheter aortic valve implantation procedure: **a** Wire insertion into the left anterior descending artery **b** Pigtail catheter in the left ventricle **c** Myval insertion **d** Myval positioning **e** Myval deployment **f** Aortic root angiography after Myval deployment **g** Final result—transcatheter aortic valve implantation-in-transcatheter aortic valve implantation

The 23 mm Myval transcatheter heart valve (THV) was mounted onto its delivery system and advanced over a stiff guidewire across the degenerated CoreValve into the LV. Under fluoroscopic guidance, the THV was carefully aligned within the existing prosthesis to minimize the risk of paravalvular leak or coronary obstruction. Deployment was achieved by balloon inflation during rapid ventricular pacing. Aortic root angiography immediately postdeployment confirmed optimal valve positioning and function (Fig. 1), with preserved flow in both the left main coronary artery (LMCA) and right coronary artery (RCA). After a 10-minute observation period, the coronary guidewire was removed, and the femoral access site was closed using a percutaneous closure device.

However, within 30 minutes postprocedure, the patient developed acute hypotension and cardiogenic shock. Urgent transthoracic echocardiography in the catheterization lab revealed severe global hypokinesia with a near absence of cardiac contractility. Emergency left femoral arterial access was obtained, and a 6 F Judkins Left (JL) guiding catheter was used to cannulate the LMCA through the struts of the CoreValve. A coronary guidewire was successfully navigated and parked in the left circumflex artery (LCX).

**Fig. 2:**
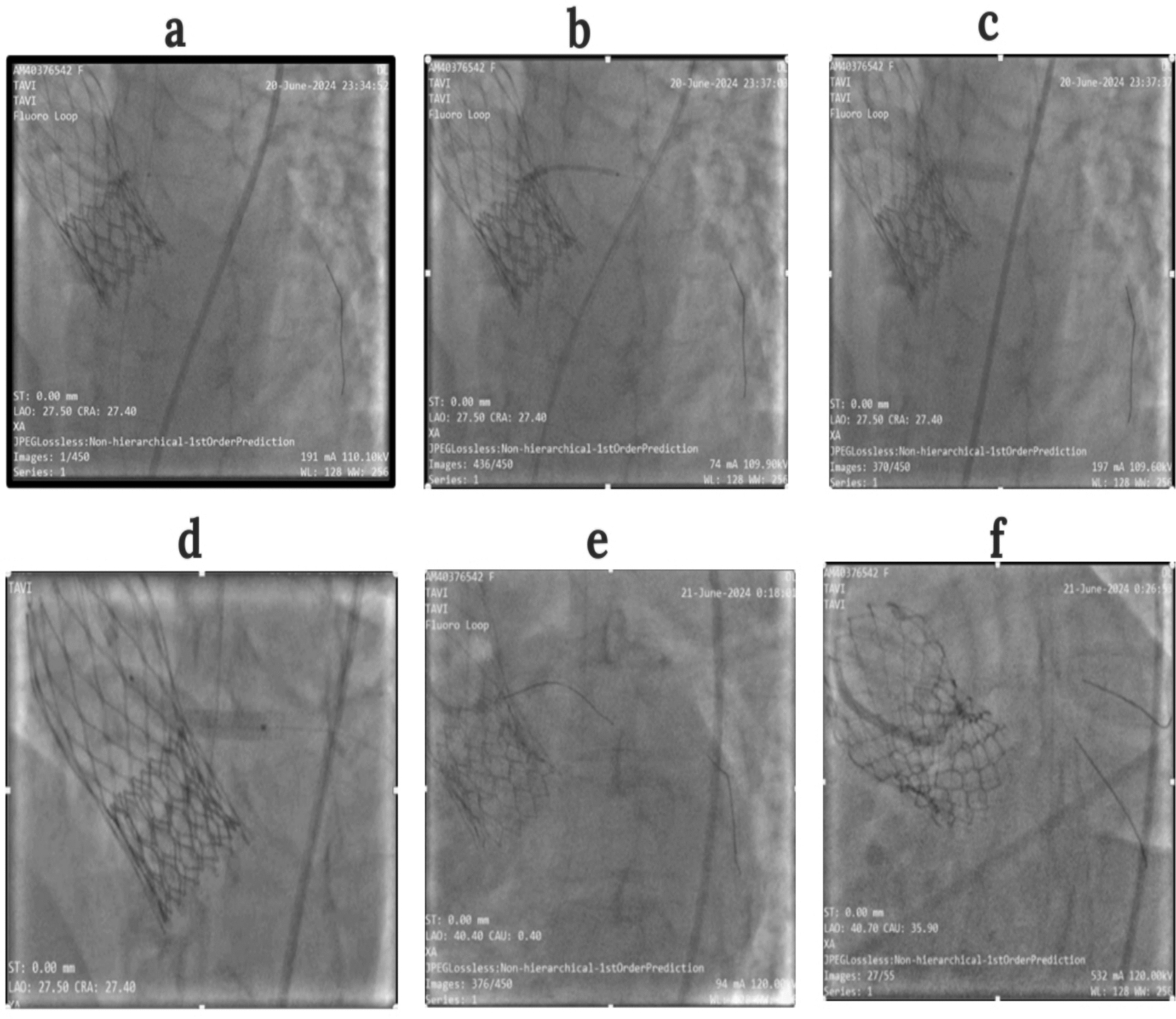
Chimney stenting technique: **a** Pre-dilatation in the left main coronary artery **b**, **c** Stent positioning and deployment in the left main coronary artery to the left anterior descending artery through valve **d** Post dilatation in the left main coronary artery ostium **e** Second wire insertion in left coronary **f** Left coronary angiography in left anterior oblique/caudal view of the left main coronary artery ostium

Initial attempts to advance a coronary stent into the LMCA were unsuccessful. Therefore, balloon pre-dilation was performed to facilitate stent delivery. Subsequently, a 4.0 × 28 mm Synergy drug-eluting stent was deployed as bailout LMCA stenting, resulting in a chimney configuration extending from the LMCA into the LCX to maintain coronary patency. Post-dilation was performed using a 5.0 × 8 mm non-compliant (NC) balloon to ensure optimal stent expansion and apposition. Coronary angiography post-stenting and post-dilation confirmed appropriate stent positioning and adequate flow restoration (Fig. 2).

Following the intervention, the patient’s hemodynamic parameters gradually stabilized, and echocardiography demonstrated restored cardiac contractility. The final angiographic assessment confirmed successful THV deployment and patent coronary arteries. Right femoral angiography ruled out access site complications. Postprocedural echocardiography showed immediate improvement in left ventricular ejection fraction (LVEF: 55%).

## Discussion

While TAVI is frequently technically successful and uneventful, there are instances where the transcatheter heart valve (THV) fails to deliver optimal results particularly in the presence of significant aortic regurgitation. In such scenarios, a bailout of TAVI-in-TAVI can serve as an effective therapeutic strategy. However, these procedures can pose clinical challenges, especially when the aortic regurgitation is substantial but not hemodynamically critical. Most existing literature focuses on elective TAVI-in-TAVI interventions, which are typically performed weeks, months, or even years after the initial TAVI rather than as an emergency response to acute valve failure during the index procedure [1].

This case highlights the successful execution of a TAVI-in-TAVI procedure. Although repeat transcatheter aortic valve implantation is emerging as a promising strategy for managing degenerated THVs, current evidence remains limited regarding the optimal procedural techniques and valve designs that offer the best long-term outcomes [2]. Such complex interventions have identified key peri-procedural challenges such as prosthesis malposition, coronary flow obstruction, and elevated postprocedural transaortic gradients. These potential complications underscore the importance of meticulous preprocedural planning, individualized device selection, and precise procedural execution to ensure patient safety and success [3].

Following comprehensive evaluation by the heart team, a TAVI-in-TAVI procedure was planned using the Myval balloon-expandable valve. The decision was guided by several key design attributes of the Myval THV: the small-cell configuration in its lower portion enhances radial strength, while its compact frame and low skirt height help minimize neo-skirt formation and reduce the risk of coronary artery occlusion. Moreover, the valve’s external polyethylene terephthalate (PET) skirt provides superior sealing, effectively reducing the likelihood of paravalvular leaks [4].

Coronary artery occlusion, though relatively uncommon, remains a serious and potentially life-threatening complication during valve-in-valve (ViV) TAVI. The risk is particularly elevated in cases involving stentless surgical bioprostheses or bioprosthetic valves with externally mounted leaflets or when the virtual transcatheter valve-to-coronary (VTC) distance is less than 4 mm. Preemptive coronary protection strategies such as the chimney technique may be employed to prevent such events. ViV TAVI in the context of degenerated surgical bioprosthetic valves is inherently complex and demands meticulous preprocedural planning, including detailed anatomical assessment and risk stratification, to anticipate and manage potential complications effectively [5].

Although often used interchangeably, it is important to distinguish between preemptive chimney stenting and bailout stenting resulting in a chimney configuration. In classical chimney technique, the stent is prepositioned and deployed simultaneously with valve implantation to ensure uninterrupted coronary perfusion. In contrast, our case involved planned coronary protection with guidewire placement, but the stent was deployed only after LMCA flow compromise was identified, constituting bailout LMCA stenting. This highlights the value of anticipatory guidewire positioning in high-risk anatomies, allowing for rapid bailout intervention if needed [6].

In this case, the patient was identified as being at elevated risk for coronary artery occlusion, primarily involving the left main coronary artery a common site for such complications. A pre-emptive coronary protection strategy was employed using the chimney technique to mitigate this risk. This approach involves the placement of a stent to create a channel that preserves coronary flow and perfusion in the event of occlusion during transcatheter valve deployment. Although the chimney technique is an established bailout strategy in TAVI, its utilization remains relatively rare, reported in only 0.5–2.2% of cases. However, studies have shown that proactive implementation of this technique can significantly reduce the incidence of major adverse cardiovascular events [5]. An alternative strategy for preventing coronary obstruction in high-risk TAVI cases is the bioprosthetic or native aortic scallop intentional laceration to prevent iatrogenic coronary artery obstruction (BASILICA) technique, which involves intentional laceration of the aortic leaflet to create a flow channel to the coronaries [7]. However, in this case, BASILICA was not pursued due to limited institutional experience with the procedure and the feasibility of guidewire-based coronary protection with bailout stenting as a preferred approach.

The patient was subsequently discharged in a hemodynamically stable condition. This case underscores the clinical advantages of using a balloon-expandable valve in valve-in-valve TAVI procedures. It also represents one of the few reported cases involving using a Myval valve in a TAVI-in-TAVI setting, demonstrating the feasibility, safety, and efficacy of this approach in managing degenerated transcatheter heart valves with heightened anatomical complexity.

## Conclusion

To the best of our knowledge, this case report highlights the first use of the Myval THV in a TAVI-in-TAVI procedure in East India. The successful outcome demonstrates that TAVI-in-TAVI using the Myval THV is feasible, safe, and effective. The procedure led to excellent hemodynamic and clinical results, showcasing the advancements in THV technology and its application in complex clinical scenarios. This case sets a significant precedent for future TAVI-in-TAVI interventions, demonstrating the evolving capabilities of new-generation balloon-expandable valves in addressing the needs of patients with degenerative THVs and also underscores the potential for wider application of the Myval THV in similar cases.

## Data Availability

All relevant clinical data supporting the findings of this case report are included in the article. Additional information is available from the corresponding author upon reasonable request.

## References

[1] Giordano A, Corcione N, Barbanti M, Costa G, Dipietro E, Amat-Santos I, *et al*. Features and outcomes of bailout repeat transcatheter aortic valve implantation (TAVI): the bailout acute TAVI-in-TAVI to lessen events (BATTLE) international registry. Clin Res Cardiol. 2023;113:1–7. 37294310 10.1007/s00392-023-02239-8 PMC10808138

[2] Procedural and clinical outcomes of patients undergoing a TAVI in TAVI procedure: rationale and design of the multicentre, prospective, observational ReTAVI registry—Parma—2024—European Journal of Clinical Investigation—Wiley Online Library. 10.1111/eci.14241 10.1111/eci.14241 38767226

[3] Voudris V, Iakovou I, Kosmas I, Sbarouni E. Repeated transcatheter aortic valve implantation for the treatment of a degenerated transcatheter aortic valve implantation valve (valve-in-valve technique): a case report. Eur Heart J Case Rep. 2020;4:1–6. 34109287 10.1093/ehjcr/ytaa256 PMC8183662

[4] Mihailovič PM, Žižek D, Vitez L, Holc P, Klokočovnik T, Bunc M. Case report: a complex case of valve-in-valve TAVI and left bundle branch pacing for severe aortic regurgitation with partially corrected type A aortic dissection and low ejection fraction. Front Cardiovasc Med. 2023. 10.3389/fcvm.2023.1206811/full. 37636302 10.3389/fcvm.2023.1206811 PMC10449538

[5] Bunc M, Vitez L, Ussia GP. Valve-in-Valve transcatheter aortic valve implantation with acute left and right coronary artery occlusion: a case report. J Med Cases. 2022;13:172–7. 35464332 10.14740/jmc3868 PMC8993451

[6] Mercanti F, Rosseel L, Neylon A, Bagur R, Sinning J-M, Nickenig G, *et al*. Chimney stenting for coronary occlusion during TAVR. JACC Cardiovasc Interv. 2020;13:751–61. 32192695 10.1016/j.jcin.2020.01.227

[7] Khan JM, Greenbaum AB, Babaliaros VC, et al. The BASILICA trial. КАРДИОЛОГИЯ УЗБЕКИСТАНА. 2019;12:1240–52. 10.1016/j.jcin.2019.03.035 PMC6669893 31202947

